# High-Speed Continuous Wavelet Transform Processor for Vital Signal Measurement Using Frequency-Modulated Continuous Wave Radar

**DOI:** 10.3390/s22083073

**Published:** 2022-04-16

**Authors:** Chanhee Bae, Seongjoo Lee, Yunho Jung

**Affiliations:** 1 Department of Smart Air Mobility, Korea Aerospace University, Goyang-si 10540, Korea; zorg0909@kau.kr; 2 Department of Information and Communication Engineering and Convergence Engineering for Intelligent Drone, Sejong University, Seoul 05006, Korea; seongjoo@sejong.ac.kr; 3 School of Electronics and Information Engineering, Korea Aerospace University, Goyang-si 10540, Korea

**Keywords:** frequency-modulated continuous wave (FMCW), vital signal measurement, radix-2 single-path delay feedback (R2SDF), mixed-radix multipath delay commutator (MRMDC)

## Abstract

This paper proposes a high-speed continuous wavelet transform (CWT) processor to analyze vital signals extracted from a frequency-modulated continuous wave (FMCW) radar sensor. The proposed CWT processor consists of a fast Fourier transform (FFT) module, complex multiplier module, and inverse FFT (IFFT) module. For high-throughput processing, the FFT and IFFT modules are designed with the pipeline FFT architecture of radix-2 single-path delay feedback (R2SDF) and mixed-radix multipath delay commutator (MRMDC) architecture, respectively. In addition, the IFFT module and the complex multiplier module perform a four-channel operation to reduce the processing time from repeated operations. Simultaneously, the MRMDC IFFT module minimizes the circuit area by reducing the number of non-trivial multipliers by using a mixed-radix algorithm. In addition, the proposed CWT processor can support variable lengths of 8, 16, 32, 64, 128, 256, 512, and 1024 to analyze various vital signals. The proposed CWT processor was implemented in a field-programmable gate array (FPGA) device and verified through the measurement of heartbeat and respiration from an FMCW radar sensor. Experimental results showed that the proposed CWT processor can reduce the processing time by 48.4-fold and 40.7-fold compared to MATLAB software with Intel i7 CPU. Moreover, it can be confirmed that the proposed CWT processor can reduce the processing time by 73.3% compared to previous FPGA-based implementations.

## 1. Introduction

The invasive measurement of human vital signals severely limits independence and mobility and may cause pain owing to the electrodes of patches and sensors. Moreover, attachment is difficult in burn victims or injured patients [1,2,3]. Owing to this problem, long-term monitoring during sleep and healthcare is difficult to accomplish using this technique [4,5]. Non-invasive measurement is an attractive alternative to vital signal measurement to overcome this limitation. There are various approaches such as depth cameras, infrared thermal sensors, and radar. For instance, depth cameras can estimate movement of the body’s trunk during breathing [6]. Infrared thermal sensors can show the temperature profile associated with the inspiration and expiration phases [7], and radar sensors can detect Doppler shifts produced by the movement of the breathing and beating heart [8,9,10,11,12,13,14,15]. Radar sensors are widely applied in vital signal monitoring because of their ability to penetrate nonmetallic obstacles and their insensitivity to environmental factors such as light and temperature [16,17]. In addition, they can be implemented with low power and in a small area [18,19,20,21,22,23].

Radar-based vital signal processing requires time-frequency analysis to detect non-stationary signals. A continuous wavelet transform (CWT) can obtain the time-frequency representation of an input signal with a high time and frequency resolution [14,24,25,26,27]. However, vital-signal measurement using time-domain CWT has the issue of long processing time owing to computational complexity, which occurs by convolution operations [28]. On the other hand, the frequency-domain processing of the CWT is appropriate for real-time implementation because it requires less computation. The CWT operation in the frequency domain requires fast Fourier transform (FFT) and inverse FFT (IFFT) operations for the transition between time and frequency domains. A hardware CWT accelerator is required to accelerate these operations [29,30].

The frequency-domain CWT uses the characteristics of FFT to replace the complex convolution in the time domain with simple multiplication in the frequency domain. FFT processors can be implemented using a single butterfly architecture, pipeline architecture, and parallel architecture. The pipeline architecture has a good tradeoff between hardware area and speed [31,32]. The pipeline architecture is divided into single-path delay feedback (SDF) and multipath delay commutator (MDC) architectures. The SDF architecture is simple, as it provides the lowest number of non-trivial multiplications in a single-channel FFT [33]. However, when using multiple channels, it has the disadvantage of linearly increasing hardware complexity in proportion to the number of channels, because it should be implemented in each channel. In the case of multichannel FFT, it is known that the MDC architecture can be implemented in a smaller area than the SDF architecture [34].

The structure of the proposed CWT processor is as follows: The FFT module for transitioning the input data to the frequency domain is designed as a radix-2 SDF (R2SDF) architecture to minimize the area. For high-speed operation, the multiplier and IFFT module after the FFT are configured in a four-channel structure to reduce the processing time from the repeated operation. The IFFT module is designed with a mixed-radix MDC (MRMDC) architecture to decrease the number of non-trivial multiplications to perform high-speed operations while minimizing the module area. The length of the vital signal can vary depending on the measurement target, such as the electroencephalogram (EEG), respiration, and heart rate [23,24,25]. Therefore, the FFT/IFFT modules of the R2SDF and MRMDC architectures are designed to support variable lengths of 8, 16, 32, 64, 128, 256, 512, and 1024, so they can be applied to various applications. The novel contribution of this work is that the proposed CWT processor proved to be effective for real-time vital signal measurement requiring high speeds and flexibility in the wavelet type, signal length, and number of daughter wavelets.

The remainder of this paper is organized as follows: Section 2 describes the CWT algorithm and basic hardware architecture. Section 3 describes the hardware architecture of the proposed CWT processor. Section 4 presents the design and implementation of the proposed CWT processor. Finally, Section 5 concludes the paper.

## 2. CWT Algorithm and Hardware Architecture

### 2.1. CWT Algorithm

**Figure 1:**
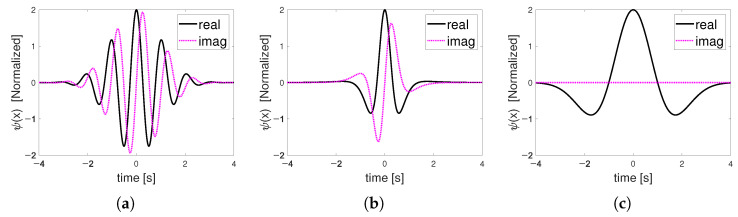
Time-domain wavelet function: (**a**) Morlet; (**b**) Paul; (**c**) Mexican hat.

**Figure 2:**
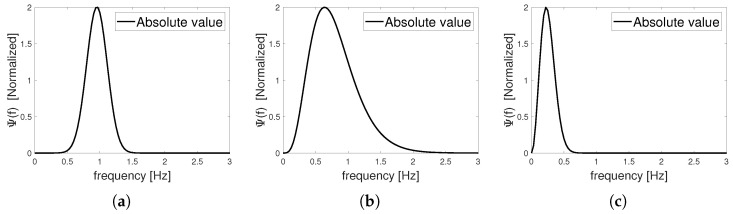
Frequency-domain wavelet function (**a**) Morlet; (**b**) Paul; (**c**) Mexican hat.

CWT uses a convolution operation to compare the input signal to a daughter wavelet, which is a compressed or stretched version of the mother wavelet. Wavelets are signals that oscillate around zero and behave like bandpass filters. Figure 1 and Figure 2 show Morlet, Paul, and Mexican hat wavelets in the time and frequency domain, respectively.

The scaling factor is used to compress or stretch the mother wavelet to produce a daughter wavelet. Low scales compress the wavelet in the time domain and effectively analyze the high-frequency components in the signal. On the other hand, high scales stretch the wavelet and are suitable for analyzing low-frequency components. Mathematically, CWT is defined in Equation (1) in the time domain.

(1) $$W_{\psi }\left(s,t\right)=\int_{-\infty }^{\infty }\psi^{*}\left(\frac{\tau -t}{s}\right)x\left(\tau \right)d\tau$$

where x(t) is the input signal, $\psi \left(t\right)$ is the mother wavelet, (·)* is a complex conjugate, and *s* is the scale factor.

The CWT can select an appropriate wavelet according to the shape of the signal to be analyzed; therefore, it has good applicability to the measurement of vital signals where the shape of the signal is important. The two-dimensional stacking of absolute values obtained through the calculation of Equation (1) using daughter wavelets of various scales is called a scalogram, and time-frequency analysis can be performed using this. Compared with the short-time Fourier transform (STFT), the CWT provides a multi-resolution measurement between the time and frequency resolutions. In addition, compared with the Fourier transform, it has the advantage that a higher frequency resolution can be obtained even with a shorter time window [14]. However, CWT operations are challenging to apply in real-time applications owing to their high computational complexity. The computational amount of the time-domain CWT is proportional to the square of the signal length owing to the convolution operation. This complexity problem can be solved using the relation defined in Equation (2).

(2) $$x\left(t\right)*y\left(t\right)\;\Leftrightarrow X\left(w\right)\times Y\left(w\right)$$

where x(t) and y(t) denote time-domain signals, and X(w) and Y(w) denote frequency-domain signals. The convolution operation in the time domain can be treated as a multiplication in the frequency domain and vice versa. In other words, CWT can significantly reduce the computational complexity by changing the convolution operation to a multiplication operation after transitioning the time-domain input signal and wavelet to the frequency domain. This is called FFT-based CWT, which enables the CWT to be operated in real time, even in applications using multiple scales.

### 2.2. FFT-Based CWT Hardware Architecture

**Figure 3:**
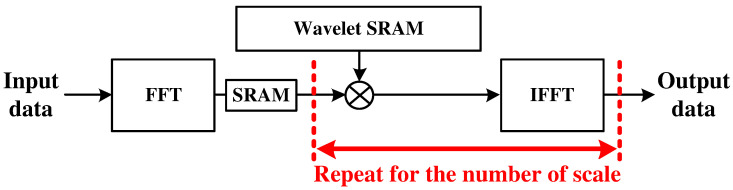
Date flow of FFT-based CWT processor.

The FFT-based CWT is mainly performed in three steps: (1) FFT operation for the input signal, (2) multiplications between the wavelet and the FFT result of the input signal, and (3) IFFT operation of the multiplication results. Figure 3 shows the data flow of the basic FFT-based CWT operations. In the figure, the FFT operation is performed once, and multiplication and IFFT are repeated according to the number of scales used.

When the FFT and IFFT modules are implemented with a single butterfly architecture based on the radix-2 algorithm, $N{log}_{2}N$ cycles are required to compute the FFT and IFFT, where *N* denotes the FFT length. In previous studies, a CWT processor was implemented using a single butterfly architecture based on the radix-4 algorithm [29,30]. This radix-4 based architecture provides a higher throughput than the radix-2 based architecture. However, owing to the limitations of the single-butterfly architecture, many cycles are still required. This makes it unsuitable for applications requiring high-speed processing, such as monitoring respiration or heartbeat rate, indicating sleep apnea and congestive heart failure.

Among the pipeline architectures of the FFT, R2SDF has an *N* computation cycles. If FFT and IFFT operations are performed with R2SDF, a total of 3N+MN cycles are required for the CWT operation. This is a very short computation cycle compared with a single butterfly architecture. However, considering the iterations of the IFFT operation, it is difficult to expect a good acceleration effect owing to the one-channel operation. For better acceleration, it is desirable to suppress the number of iterations using the MDC architecture specialized for multichannel operation. The CWT processor proposed in this study is accelerated by reducing the number of iterations by performing multichannel multiplication and IFFT operations. The architecture of the proposed processor is described in Section 3.

## 3. Hardware Architecture of the Proposed CWT Processor

**Figure 4:**
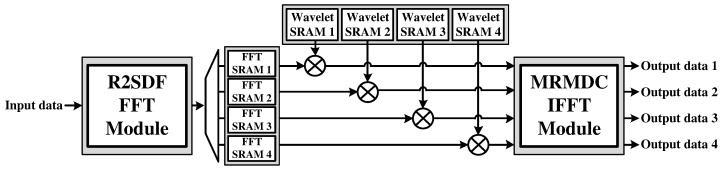
Hardware architecture of the proposed CWT processor.

Figure 4 shows the hardware architecture of the proposed CWT processor for high-speed vital signal processing. It consists of an R2SDF FFT module for the FFT of the input signal, four FFT static random access memories (SRAMs) for data reordering, and dividing the FFT result into four-channel, four wavelet SRAMs with loaded daughter wavelet values in the frequency domain, four multipliers, and an MRMDC IFFT module for the four-channel IFFT.

**Figure 5:**
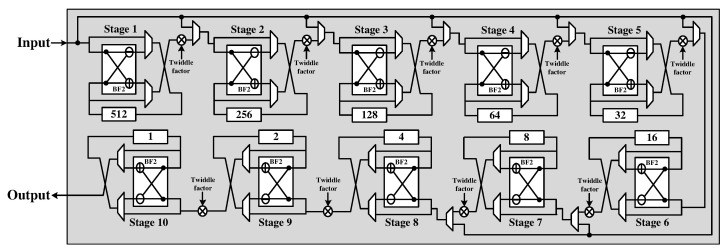
Hardware architecture of R2SDF FFT module.

Figure 5 shows the hardware architecture of the R2SDF FFT module, which is known to satisfy the trade-off between the throughput and hardware area for a one-channel FFT. The module can support 8, 16, 32, 64, 128, 256, 512, and 1024-point lengths by selecting the input signal for the operation in each stage.

**Figure 6:**
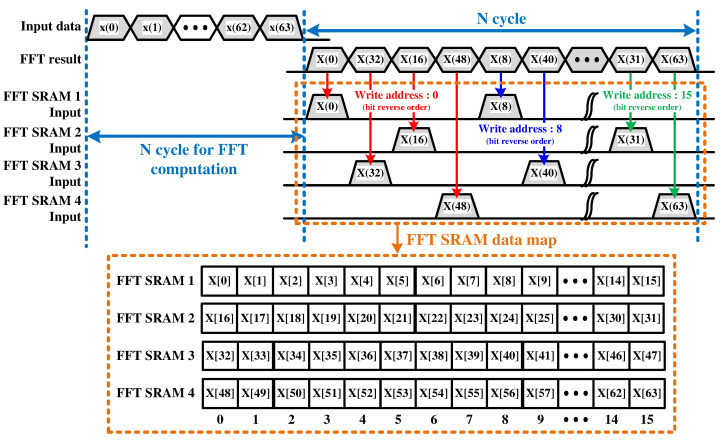
Timing diagram of FFT SRAM writing process.

As mentioned, the SDF architecture has the disadvantage of linearly increasing the hardware complexity in proportion to the number of channels. In the case of a four-channel FFT operation, one radix-4 MDC (R4MDC) architecture is implemented with a smaller area than the four R2SDF architectures [35]. However, if the existing MDC architecture is applied, there is a problem that the overall computational latency and the processor area will also increase. This is because of the many delay elements of the first data mapping module (DMM) that align the data from the input stage [36]. The first DMM for the 1024-point FFT has 3072 delay elements, which corresponds to a memory size of approximately 12.3 KB based on 32-bit complex data. To reduce this resource, the proposed CWT processor imitates the data alignment process of the first DMM using four FFT SRAMs to store the FFT results. The proposed processor removes the first DMM by storing the FFT results in four FFT SRAMs and performing the aligning function for MDC IFFT. Figure 6 shows the timing diagram for storing the 64-point FFT results in the four FFT SRAMs and the final memory map of the FFT SRAMs.

**Figure 7:**
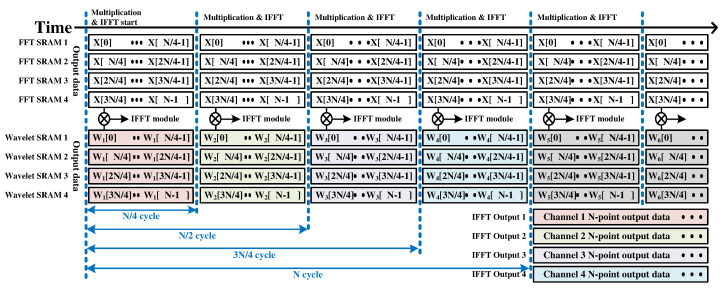
Timing diagram of multiplication and IFFT.

After the FFT result is written to all four SRAMs, four-channel multiplication is performed. For multiplication, the proposed processor has four wavelet SRAMs that can load daughter wavelets in the frequency domain. At most, 24 wavelet signals with 1024 points can be loaded. The output values of the four FFT SRAMs and wavelet SRAM are multiplied and used as the input of the IFFT module. Figure 7 shows the timing diagram of the proposed processor’s four-channel multiplication and IFFT operations. In Figure 7, FFT SRAM is the output of FFT SRAM, and wavelet SRAM denotes the output of wavelet SRAM.

**Figure 8:**
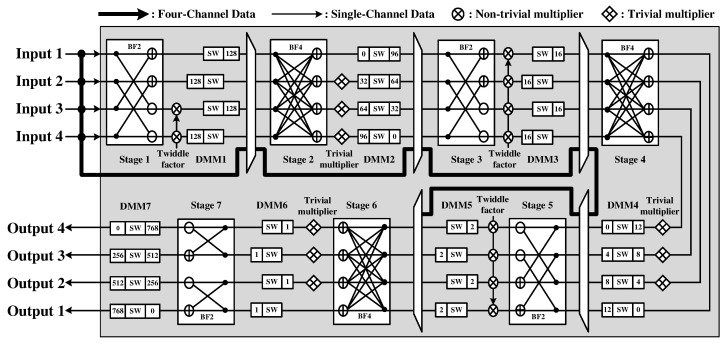
Hardware architecture of MRMDC IFFT module.

The four-channel IFFT module, in which the first DMM has been removed, is designed using the MRMDC architecture. Compared with the R4MDC architecture, the MRMDC architecture has a smaller area because some non-trivial multipliers can be replaced with trivial multipliers [37]. Unlike the radix-4 algorithm, which supports variable lengths limited to a power of 4, it can support various variable lengths, such as the radix-2 algorithm [38,39]. The IFFT module is designed to support 8, 16, 32, 64, 128, 256, 512, and 1024 points of variable length data by selecting input signals from each stage, such as the R2SDF FFT module within the same processor. Figure 8 shows the MRMDC IFFT module of the proposed CWT processor.

When the input data and wavelet data have *N*-point length CWT operations using *M* scales, there are 3N+(M/4)N computation cycles. In this case, *M* is a multiple of 4. Because 3N+MN cycles are required by the R2SDF(FFT)–R2SDF(IFFT) architecture, it can be confirmed that the proposed processor can support higher-speed CWT operation compared to the other aforementioned architectures. Because the proposed CWT architecture uses multichannel multiplication and IFFT operations, it can reduce the number of iterations. If the number of scales to be used increases, the number of iterations also increases. Therefore, there will be a more significant gap in the total number of computation cycles between the proposed CWT architecture and other architectures. Therefore, the proposed CWT processor can maintain high-speed operation, even in applications that use many scales.

## 4. Implementation Results

The proposed CWT processor was designed using the Verilog hardware description language (HDL) and implemented on a Xilinx Zynq UltraScale+ field programmable gate array (FPGA) device [40]. The CWT processor could process at a maximum operating frequency of 302 MHz. Table 1 summarizes the resource usage of the proposed CWT processor. The proposed CWT processor was implemented using 89,941 look up tables (LUTs), 108,598 flip flops (FFs), and 92 digital signal processors (DSPs).

Table 2 summarizes the computation cycles and processing time required for the proposed CWT processor when using four scales and 24 scales for inputs using 512-point and 1024-point length signals. When the operating frequency is set to 302 MHz, the processing time of a 512-point length using four scales is 7 μs, and the 1024-point length using 24 scales is 31 μs.

**Figure 9:**
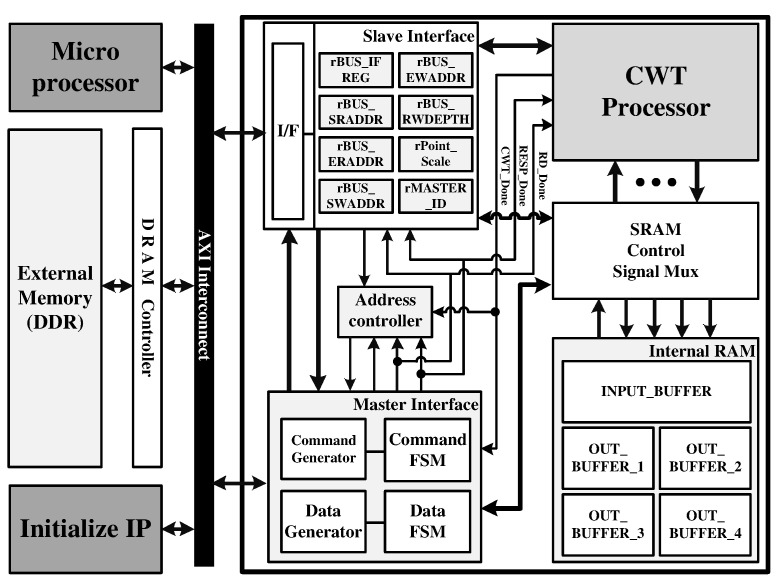
FPGA platform configuration for the verification of the proposed CWT processor.

**Figure 10:**
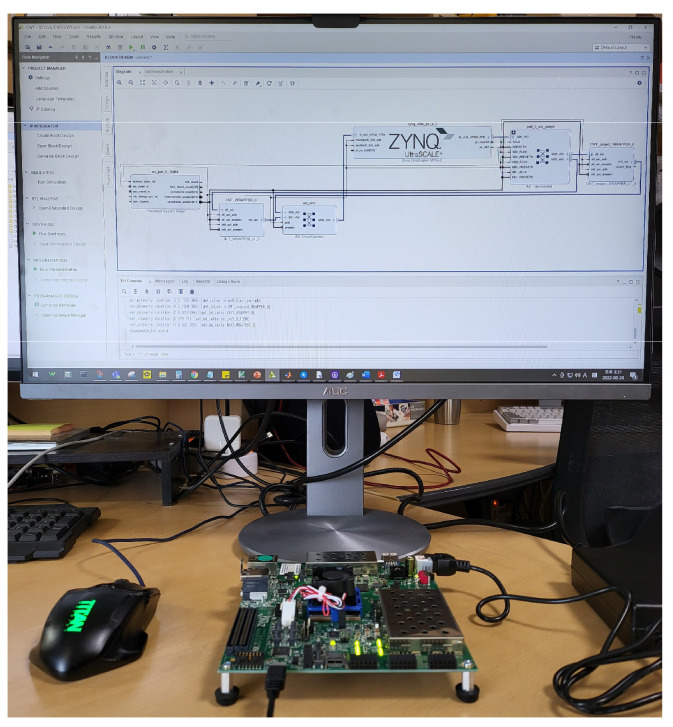
Verification environment using FPGA platform.

The proposed CWT processor was configured on the FPGA-based verification platform using the advanced extensible interface (AXI) bus interface, as shown in Figure 9. The verification platform consists of the proposed CWT processor, external double data rate (DDR) memory, AXI master interface for data load and store in DDR, and AXI slave interface for interaction between the micro-processor (MP) and the CWT processor by setting the predefined registers. First, input data and wavelet data for the verification of the CWT processor are loaded in external DDR memory. In addition, several parameters such as the FFT-point length and the number of wavelets are set by MP through an AXI slave interface. When MP initiates the CWT operation, the proposed CWT processor reads the wavelet data and input data and stores them in four wavelet SRAMs and input buffer, respectively. After the CWT operation is completed, the results are stored in external DDR memory through an AXI master interface. Figure 10 shows the verification environment using the FPGA platform.

**Figure 11:**
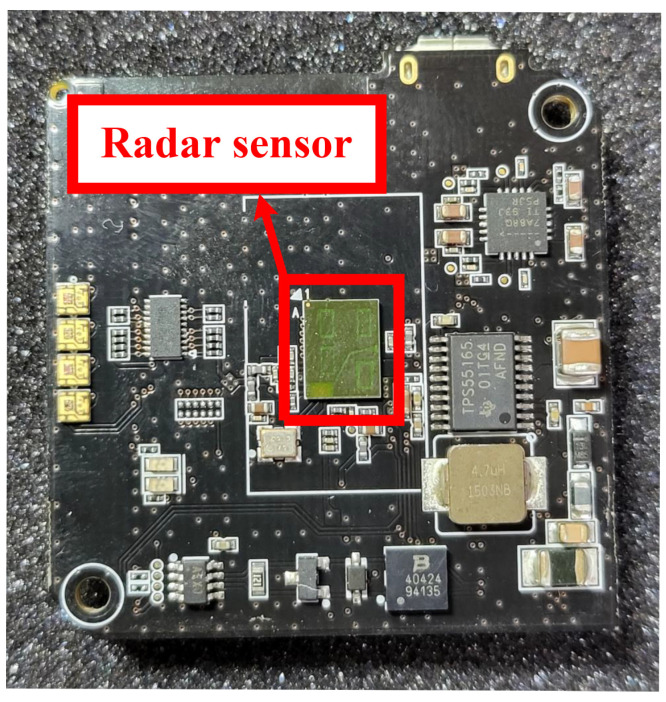
Infineon’s FMCW radar sensor for the vital signal measurement.

To verify the vital signal measurement function of the proposed CWT processor, Infineon’s BGT60TR13C frequency modulated continuous wave (FMCW) radar sensor with a center frequency of 60 GHz was used [41], as shown in Figure 11. The FMCW radar signal was irradiated to the subject’s chest to acquire Doppler shifts of the chest due to heartbeat and respiration. Table 3 shows the main parameters of the FMCW radar sensor.

**Figure 12:**
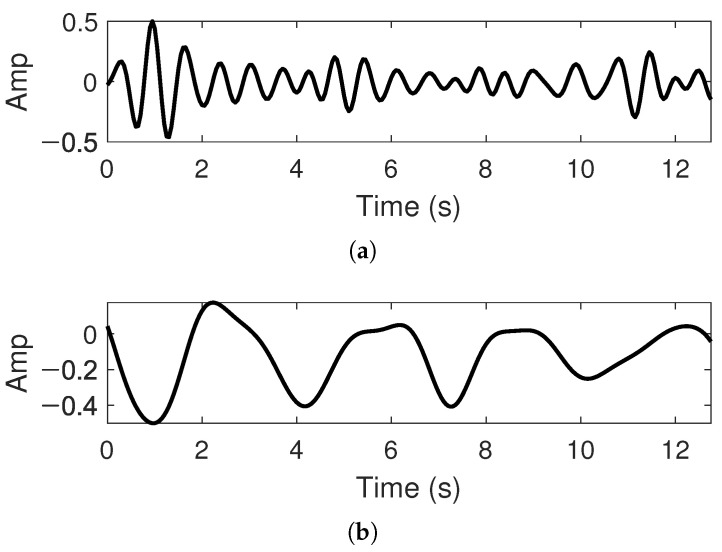
The vital signals for the verification of the proposed CWT processor: (**a**) heartbeat; (**b**) respiration.

Figure 12 shows the heartbeat and respiration signals acquired for the verification of the proposed CWT processor. The data of each signal were sampled at 20 Hz and divided to have a length of 512-point (12.8 s).

A morlet wavelet was used as a mother wavelet for the CWT analysis of vital signals. Since each vital signal has a different main frequency component, a different scale should be used for the analysis. A total of 24 daughter wavelets of scale from 5.8 to 28.7 were used for the heartbeat signal, which corresponds to a frequency range of from 0.7 to 3.44 Hz. Since the respiration signal has a lower frequency component than the heartbeat signal, a larger scale should be used. A total of 20 scales ranging from 23.3 to 86.9 with 0.23 to 0.86 Hz were used to analyze the respiration signal. The heartbeat, respiration signal, and daughter wavelet were oversampled to 1024-points length with zero padding for the smoother CWT results.

**Figure 13:**
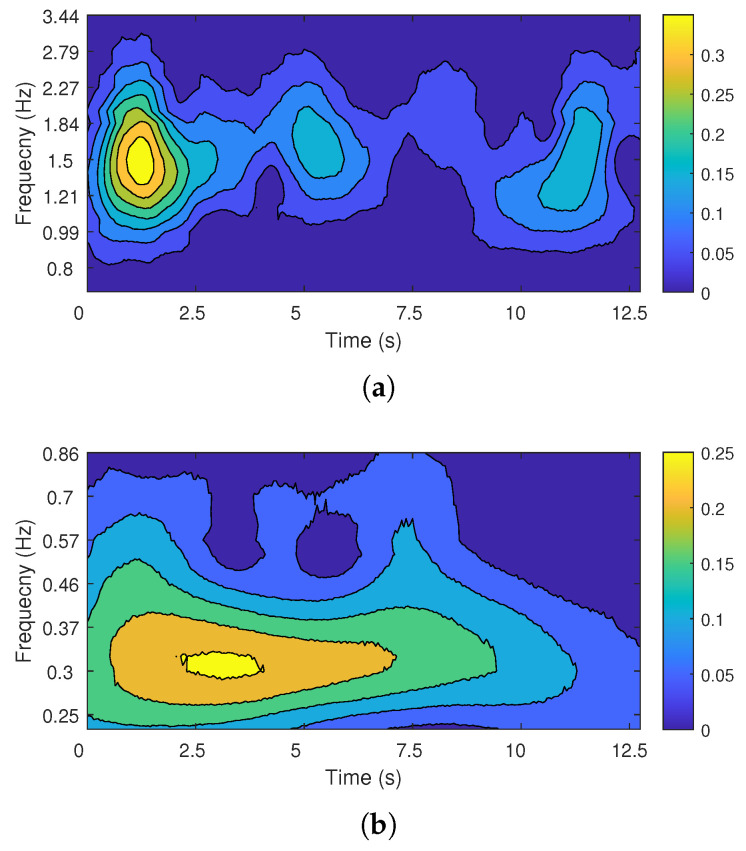
The CWT scalogram from the proposed CWT processor: (**a**) heartbeat; (**b**) respiration.

**Figure 14:**
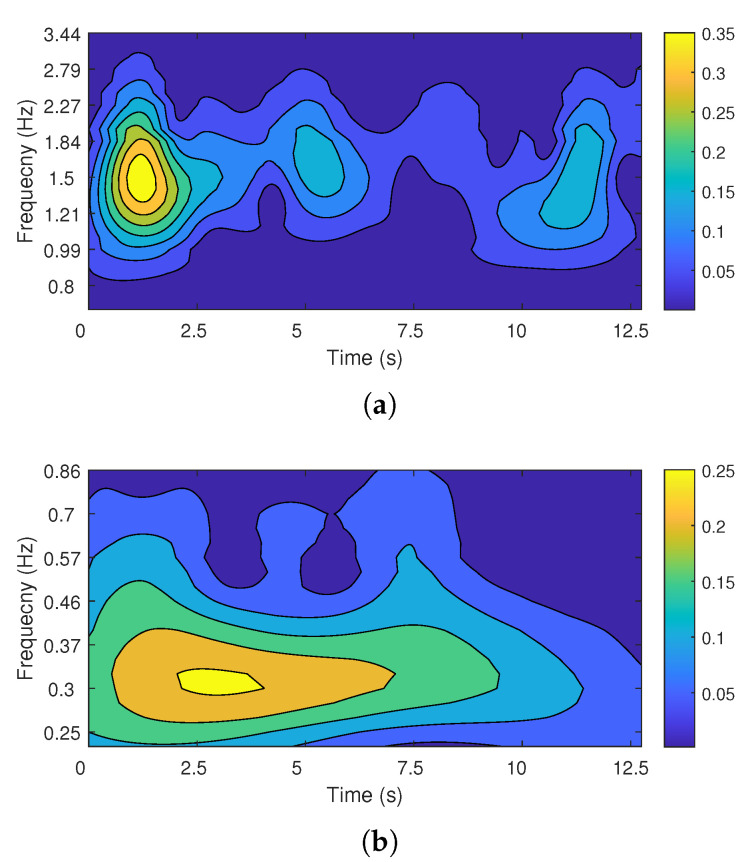
The CWT scalogram from MATLAB: (**a**) heartbeat; (**b**) respiration.

The vital signal acquired from the FMCW radar sensor was used as the input of the proposed CWT processor, and the result of the CWT processor was compared with that of the CWT function implemented in software (MATLAB R2020a [42]). Figure 13 shows the scalograms from the proposed CWT processor, and Figure 14 shows the scalograms from MATLAB software. The peak frequency and temporal location of the scalogram from the proposed CWT processor and MATLAB software were analyzed, and the same evaluation results were obtained: The heartbeat signal has a peak frequency of 1.5 Hz at 1.2 s, and the respiration signal has a peak frequency of 0.3 Hz at 3.1 s. The slight difference between Figure 13 and Figure 14 is due to the different number systems used. The proposed CWT processor uses a 16-bit fixed-point number system, while MATLAB software uses a 64-bit floating-point number system. The proposed CWT processor has a signal-to-quantization noise ratio (SQNR) of about 32.1 dB in the effective data range.

The processing time for the proposed CWT processor was compared with that for the MATLAB software. The proposed CWT processor consumes 0.031 ms for the CWT operation of heartbeat signal using 24 scales, and 0.027 ms for respiration signal using 20 scales. In the case of MATLAB software using Intel i7 CPU at 3.6 GHz [43] with 32 GB RAM, about 1.5 ms and 1.1 ms were used to process heartbeat and respiration signals, respectively. These results shows that the proposed CWT processor can reduce the processing time for a CWT operation by 48.4-fold and 40.7-fold, respectively, compared to the MATLAB software. Table 4 summarizes the comparison results for the processing time between the proposed CWT processor and MATLAB software operation.

A comparison was made with previous research to confirm that the proposed CWT processor has high-speed processing capabilities. When comparing the implemented CWT processor, it excludes the implementation of a general-purpose processor (GPP), DSP, and 2-D CWT implementation [44,45,46,47]. Qasim et al. implemented a CWT processor using the radix-4 burst I/O of the Xilinx FFT core V.5 for EEG function extraction [29,30]. For the fairest possible comparison, the processing time was normalized using the $T_{norm}$ expressed in Equation (3).

(3) $$T_{norm}\;=\;\frac{16nm}{Tech}\times \frac{1024}{Signal\;length}\times \frac{24}{\#\;of\;scales}\times Processing\;Time$$

The ‘Tech’ in Equation (3) represents the CMOS process technology of the FPGA. The ‘Signallength’ of the equation is the length of input data, which affects the FFT computation time. Finally, the number of scales was normalized to reduce the effect of the number of scales used. Table 5 compares the proposed CWT processor and the CWT processor described in [30], which is more recent than that in [29].

The proposed processor can reduce the operation time of the CWT processor in reference [30] by 73.3%. In this case, the proposed processor focused on various vital signal measurements; therefore, the wavelet could not be fixed at the constant values. On the other hand, the reference processor focuses only on EEG feature extraction; therefore, the wavelet that can be used for observation is a fixed type. Consequently, the reference processor was able to reduce the number of cycles by skipping unnecessary parts in the multiplication process and increasing the maximum operating frequency. If the type of wavelet should be flexible for a variety of applications, the difference in $T_{norm}$ between the reference processor and the proposed processor will be considerable. Therefore, the proposed processor has a faster CWT processing time than the reference processor, which makes it more suitable for real-time general vital-signal measurement.

## 5. Discussion and Conclusions

This paper proposes an FFT-based CWT processor for high-speed vital-signal measurement. The proposed processor uses a pipeline FFT/IFFT architecture to satisfy the trade-off between the throughput and hardware complexity. The FFT module for one-channel input signals used an R2SDF architecture with low complexity. The IFFT module for foura-channel input signals applies an MDC architecture with a smaller area than the SDF architecture. Furthermore, it can decrease the number of non-trivial multipliers and support various input signal lengths of 8, 16, 32, 64, 128, 256, 512 and 1024-point length by using a mixed-radix algorithm. The proposed processor can support up to 24 daughter wavelets with a maximum 1024-point length. The number of loaded daughter wavelets can be controlled for use in various vital signal measurement applications. The proposed CWT processor was implemented on a Xilinx Zynq UltraScale+ FPGA device and used 89,941 LUTs, 108,598 FFs, and 92 DSPs resources at the operating frequency of 302 MHz. The processing time between the proposed CWT processor implemented in the FPGA device and MATLAB software with an Intel i7 CPU were compared, and it is confirmed that the proposed CWT processor can reduce the processing time by 48.4-fold and 40.7-fold for heartbeat and respiration from an FMCW radar sensor, respectively. Finally, the proposed processor is compared with a previous processor implemented using the FFT-based CWT algorithm. As a result, the proposed processor can reduce the CWT processing time of 1024-point signals by 94.6% compared to the previous CWT processor. In addition, it was confirmed that the processing time could be reduced by 73.3% if normalization was performed with the technology of the FPGA device used, signal length, and number of scales for a fair comparison. Thus, the proposed CWT processor proved to be effective for real-time vital signal measurement requiring high speeds and flexibility in wavelet type, signal length, and number of daughter wavelets.

## Figures and Tables

**Table 1:** Implementation results of the proposed CWT processor.

Block	No. of LUT	No. of FF	DSP Block
R2SDF	41,179	38,789	16
Complex multiplier	611	435	16
MRMDC	47,394	69,299	40
Others	757	75	0
Total	89,941	108,598	92

**Table 2:** Processing cycles and time for the design in two different ranges of wavelet scales.

Task		Cycles for 512-Point Signal (4 Scales)	Cycles for 1024-Point Signal (24 Scales)
FFT (R2SDF)	Compute	530	1060
SRAM	Writing	512	1024
IFFT (MRMDC)	Compute	531	1047
	Data output	512	6144
Total number of cycles		2085	9275
Total processing time		7 μs @302 MHz	31 μs @302 MHz

**Table 3:** Main parameters of FMCW radar sensor.

Parameter	Value
Center frequency	60 GHz
Bandwidth	5.5 GHz
Antenna gain (single TX / RX)	5 dBi
Maximum distance	15 m
FoV (half power beam width)	90°

**Table 4:** Comparison of the processing time between the proposed CWT processor and MATLAB software.

Signal	Input Data	No. of Scales	Processing Time (ms)		Reduction
	Length		MATLAB	Proposed	(Fold)
Heartbeat	1024-point	24	1.5	0.031	48.4
Respiration		20	1.1	0.027	40.7

**Table 5:** Comparison of the proposed CWT processor with the previous research results.

Work	[30]	This Work
FPGA device	Spartan-3AN	Zynq UltraScale+
Technology (nm)	90	16
Signal point (N)	1024	8/16/32/64/128/256/512/1024
No. of scale	21	24
Max. Freq (MHz) ^1^	133	302
Processing time (ms)	0.57	0.031 (max)
$T_{norm}$ (ms)	0.116	0.031

^1^ Maximum operating frequency

## Data Availability

Not applicable.
